# Sialidase and Sialyltransferase Inhibitors: Targeting Pathogenicity and Disease

**DOI:** 10.3389/fmolb.2021.705133

**Published:** 2021-07-29

**Authors:** William H. D. Bowles, Tracey M. Gloster

**Affiliations:** Biomedical Sciences Research Complex, School of Biology, University of St Andrews, St Andrews, United Kingdom

**Keywords:** sialidase, sialyltransferase, inhibition, neuraminidase, sialic acid, Neu5Ac, cancer, influenza

## Abstract

Sialidases (SAs) and sialyltransferases (STs), the enzymes responsible for removing and adding sialic acid to other glycans, play essential roles in viruses, bacteria, parasites, and humans. Sialic acid is often the terminal sugar on glycans protruding from the cell surface in humans and is an important component for recognition and cell function. Pathogens have evolved to exploit this and use sialic acid to either “cloak” themselves, ensuring they remain undetected, or as a mechanism to enable release of virus progeny. The development of inhibitors against SAs and STs therefore provides the opportunity to target a range of diseases. Inhibitors targeting viral, bacterial, or parasitic enzymes can directly target their pathogenicity in humans. Excellent examples of this can be found with the anti-influenza drugs Zanamivir (Relenza™, GlaxoSmithKline) and Oseltamivir (Tamiflu™, Roche and Gilead), which have been used in the clinic for over two decades. However, the development of resistance against these drugs means there is an ongoing need for novel potent and specific inhibitors. Humans possess 20 STs and four SAs that play essential roles in cellular function, but have also been implicated in cancer progression, as glycans on many cancer cells are found to be hyper-sialylated. Whilst much remains unknown about how STs function in relation to disease, it is clear that specific inhibitors of them can serve both as tools to gain a better understanding of their activity and form the basis for development of anti-cancer drugs. Here we review the recent developments in the design of SA and ST inhibitors against pathogens and humans.

## Introduction

**FIGURE 1:**
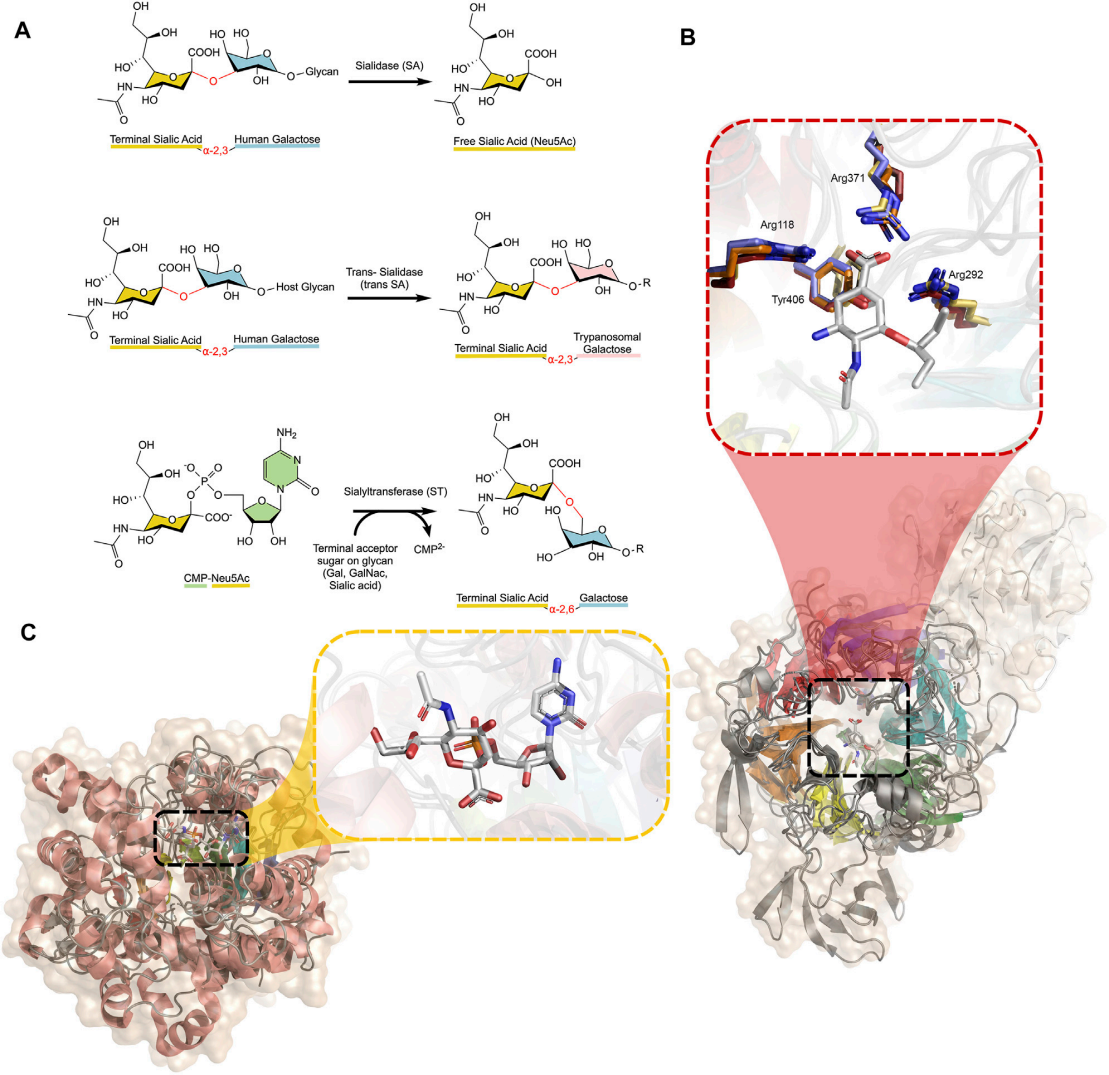
**(A)** Reactions catalysed by SAs, trans-SAs, and STs, with glycosidic bonds broken or formed shown in red between each monosaccharide. SAs and STs act to hydrolyse or create different linkages (α-2,3/α-2,6/α-2,8/α-2,9) between the monosaccharides, depending on the specific enzyme, while the trans-SA of the *Trypanosoma* family specifically acts on α-2,3 linked sialic acids **(B)** Alignment of four SAs (Influenza N1, *C. perfringens* NanI, *T. cruzi* trans-SA, *H. sapiens* NEU2) displaying the β-propeller structure with approximate six-fold symmetry highlighted in rainbow colours. The trans-SA transfer domain is shown in white. The active site is enlarged, showing oseltamivir bound in the active site. The highly conserved position of active site residues of Influenza (yellow), *C. perfringens* (orange), *T. cruzi* (red), and Human (blue) NAs, with corresponding numbering from influenza N1 (PDB IDs 3CL0, 5TSP, 2AH2, and 1VCU, respectively) (Amaya et al., 2004; Chavas et al., 2005; Collins et al., 2008; Lee et al., 2017) **(C)** Alignment of three human STs (ST3-GalNAcII, ST6-Gal-I, and ST8-SiaIII) displaying the conserved Rossman β-sheet architecture common to all STs (rainbow coloured) (PDB IDs: 6APL, 6QVT, and 5BO6, respectively). The active site is enlarged, showing CMP-Neu5Ac bound (Volkers et al., 2015; Moremen et al., 2018; Harrus et al., 2020). Figures created using PyMOL and ChemDraw.

Sialic acid is a ubiquitous carbohydrate utilized by species across the domains of life and viruses. Sialidases (SAs) and sialyltransferases (STs) (Figure 1A) are the carbohydrate processing enzymes responsible for catalysing the addition and removal of sialic acid (Neu5Ac, 1) to or from glycans attached to proteins or lipids on the cell surface. Inhibition of these enzymes is important to gain a better understanding of the biological roles played by glycans, but is also fundamental in the treatment of diseases. A number of these inhibitors were discovered several decades ago and are in clinical use, whereas others are only now being realised.

The discovery of SA inhibitors has proven vital for targeting influenza, which has historically been responsible for devastating global pandemics. Even today, influenza accounts for between 300,000–650,000 deaths and costs the global economy billions of dollars every year (Putri et al., 2018; WHO, 2018). Almost 30 years after the first influenza SA inhibitors were discovered in the 1960s, an X-ray crystal structure of influenza SA with an inhibitor bound was solved (Edmond et al., 1966; Varghese et al., 1992). This structure provided an essential scaffold on which the following structure-based drug design would be built. A wealth of papers attempting to design drugs targeting influenza SA have been published since, with many successes.

However, SAs are not only encoded in viral genomes; they are also present in bacteria and many vertebrates including mammals. Whilst these enzymes have been explored less than their viral counterparts, it is evident that there are opportunities for new applications involving SA inhibitors. For example, *Clostridium perfringens* (the causative agent of gangrene) uses three SAs to aid its pathogenicity. The human parasite *Trypanosoma cruzi* uses a trans-SA as a major virulence factor to disguise it from host defences in Chagas disease.

SAs and their ST counterparts present in mammals, including humans, have opposing roles in diseases such as diabetes, cancer and atherosclerosis. All SAs share a strikingly similar β-propeller architecture composed of antiparallel β-sheets in an approximate six-fold symmetry, despite their low primary structure sequence identity (< 26%). However, SAs all possess a conserved arginine triad and catalytic tyrosine residue in the active site (Figure 1B). STs display a conserved Rossman fold, as is typical of many glycosyltransferases (Audry et al., 2011) (Figure 1C).

The design of inhibitors against these enzymes, both based on the sialic acid scaffold and other small molecule inhibitors, is key for the development of novel therapies and potent antibiotics, antivirals, and antiparasitic drugs. This review will aim to showcase the recent progress in design and discovery of such inhibitors, with a focus on inhibitors with a sialic acid-like scaffold, and will highlight new avenues for potential future research.

### Viral Sialidase Inhibitors

**FIGURE 2:**
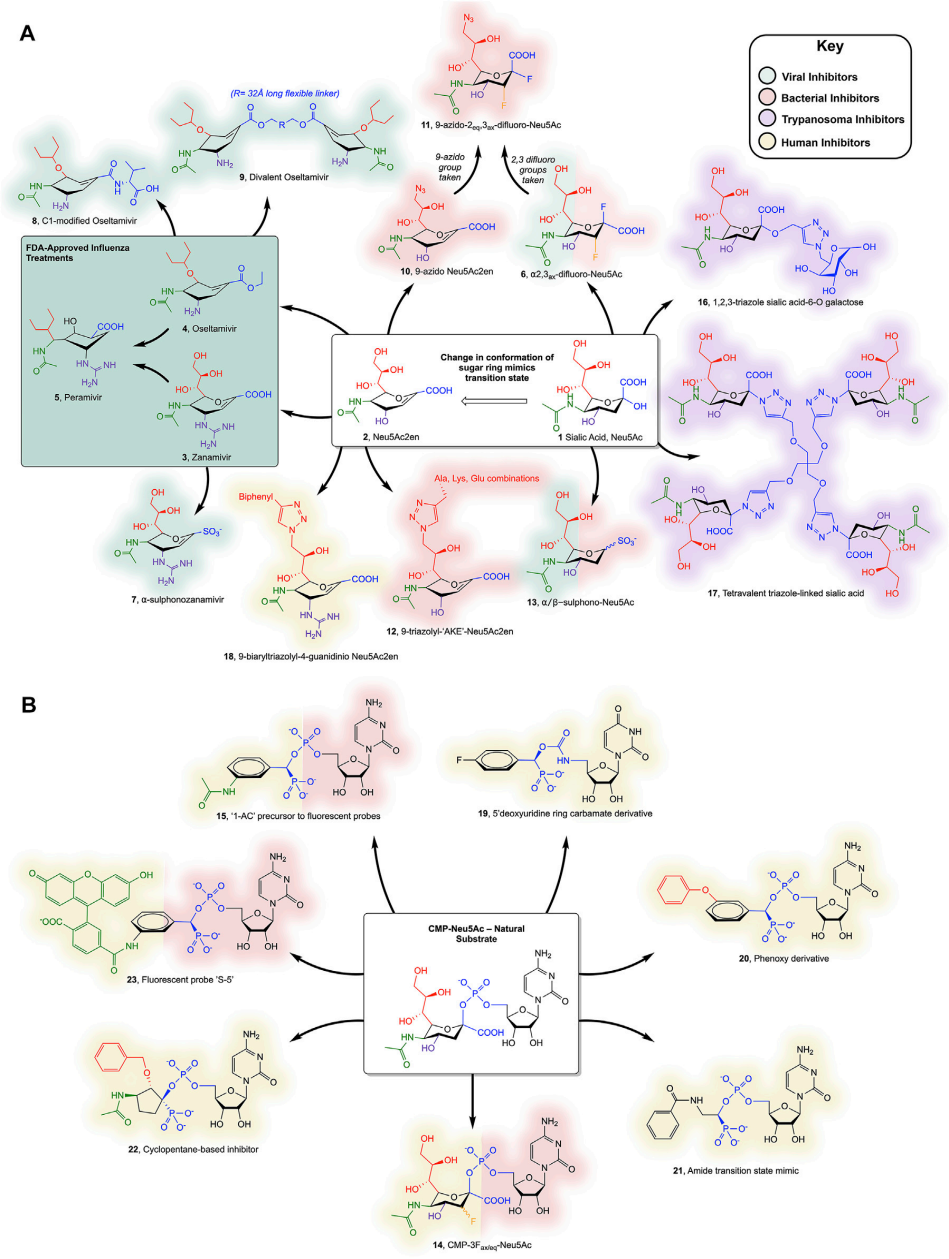
**(A)** Sialic acid- and Neu5Ac2en-derived inhibitors of SAs and **(B)** CMP-Neu5Ac-derived inhibitors of STs discussed in the text. Colours surrounding each compound represent the organism they inhibit (see key); note that compounds 6, 13, 14, and 15 inhibit more than one organism and so both representative colours are shown. Individual substituents on the Neu5Ac or Neu5ac2en ring are colour coded to highlight changes to the scaffold.

It should be noted that several viruses of the *Paramyxodivirdae* family, such as Newcastle disease virus, Parainfluenza viruses, Mumps virus, and Sendai virus, encode a haemagglutinin-sialidase protein, however this review will only consider the *Orthomyxoviridae* influenza virus SA (Taylor, 1996). It should also be noted that only one example of a viral ST exists in the literature: a myxoma virus ST3-Gal-I, which has not been extensively studied (Jackson et al., 1999; Sugiarto et al., 2011). Influenza strains are identified by the homotetrameric SA type on their surface (groups N1-N9) and employ SAs to release new viral particles from infected cells by cleaving the host surface sialic acid (Itzstein, 2007). The structures of influenza SA [which belongs to glycoside hydrolase (GH) family 34 in the CAZy database (Lombard et al., 2014)], in complex with Neu5Ac (1) and with Neu5Ac2en (2), a potent inhibitor with a double bond in the pyranose ring, were reported in 1992 (Meindl et al., 1974; Varghese et al., 1992). The Neu5Ac2en scaffold (Figure 2A), which derives its potency by mimicking the oxocarbenium ion-like transition state, was used in the structure-based drug design of two anti-influenza drugs: oseltamivir (Tamiflu™, Roche and Gilead) and zanamivir (Relenza™, GlaxoSmithKline) (3 and 4) (Itzstein et al., 1993; Kim et al., 1997). Building on their success, peramivir (5), another potent influenza SA inhibitor, was designed using computational structure-based methods (Babu et al., 2000). Each of these drugs form extensive contacts with conserved residues in the SA active site, which out-compete terminal sialic acid residues on the host glycans, thus inhibiting the release of virus progeny from infected cells. Due to viral resistance against oseltamivir, peramivir was authorized by the FDA for emergency use in the swine flu pandemic of 2009 and was fully approved in 2014 (Thorlund et al., 2011; Wester et al., 2016). Peramivir highlighted two important requirements for designing Neu5Ac2en-related inhibitors; firstly the sugar ring conformation and secondly, the position of the attached groups to maximize binding in four conserved pockets (Babu et al., 2000). Peramivir contains an unusual 5-membered ring but retains high potency as its functional groups bind in a similar way to oseltamivir and zanamivir. Due to the rapid mutation rate of influenza, a plethora of mutant influenza subtypes are becoming increasingly resistant to the clinically available drugs (Hussain et al., 2017). The ever-present threat of an influenza pandemic is of particular concern and highlights the need for pre-emptive and effective novel inhibitor design.

More recent work by Vavricka and colleagues aimed to characterize the mechanism employed by SAs in hydrolysis of the glycosidic bond in order to design covalent inhibitors (Vavricka et al., 2013). ^1^H NMR and structural data of the Tyr406Asp mutant of Influenza N2 implicated Tyr406 as the catalytic nucleophile. The addition of fluorine at C2 and C3 of sialic acid allowed formation of a covalent adduct with the hydroxyl group of Tyr406, providing the first covalent SA inhibitor, 2α,3_ax_-difluoro-Neu5Ac (6) (Vavricka et al., 2013). Two groups independently reported compounds with sulphonate modifications to Neu5Ac displaying micromolar potency against viral SAs (Hadházi et al., 2017; Vavricka et al., 2017). The same modification was applied to zanamivir, replacing the carboxyl group, which enabled interactions with the conserved Arg triad, and resulted in low-nanomolar potency against Influenza SA (Hadházi et al., 2018). Although “α−sulphonozanamivir” (7) is not more potent than zanamivir against influenza SA, it is more selective, with approximately 20-fold lower inhibition of human SA.

Zanamivir is not the only approved drug to have been modified; oseltamivir has also been modified, with the aim of designing compounds with higher potency than oseltamivir itself, whilst limiting the likelihood of resistance developing. Addition at oseltamivir’s C1, aiming to target the 430-loop (an unused cavity of influenza SA that could accommodate potential new drug interactions), has yielded an amide-linked carboxylate with comparable *in vivo* efficacy to oseltamivir (8) (Ju et al., 2018). One novel and innovative modification sees a 32 Å polyglycol addition linking two oseltamivir monomers, forming “Divalent oseltamivir” (9). The cross-linking effect allows the occupancy of more than one SA active site in the tetramer and translates to a 68-fold increase in inhibitory potency *in vitro* compared to oseltamivir (Yan et al., 2019). This strategy is currently being explored as an exciting method of increasing drug potency and selectivity (as mammalian SAs are not tetrameric), breathing new life into compounds that are prone to resistance.

### Bacterial Sialidase and Sialyltransferase Inhibitors

Multiple pathogenic bacterial species, including *Vibrio cholera*, *Corynebacterium diptheriae*, and *Streptococcus pneumoniae*, utilize SAs for pathogenicity (Severi et al., 2007). However, *Clostridium perfringens* SAs (NanI, NanJ, and NanH) are probably the best studied and understood (Corfield, 1992). *C. perfringens* is pathogenic to humans and livestock, causing gangrene, necrotizing enteritis, and food poisoning, with disastrous health and economic implications throughout the world (Freedman et al., 2016).

The hydrolysis of host cell sialic acids by SAs from *C. perfringens* is key to its virulence and is thought to play roles in pathogenesis, including destruction of the intestinal cell barriers (Cioffi et al., 2012), promoting adhesion to the cell wall by reduction of negative charges (Li et al., 2011; Lewis and Lewis, 2012; Li and McClane, 2014) and using the sugars in metabolism to out-compete other bacteria (Corfield, 1992). The final and perhaps most vital function of the *C. perfringens* SAs is the ability to potentiate *C. perfringens* toxins, however the mechanism of this is unclear (Li et al., 2015a; Theoret et al., 2018). Interestingly, the three SAs, NanI, NanJ, and NanH (which all belong to family GH33 in the CAZy database), act synergistically on the hydrolysis of complex sialic acid-containing glycans, with substrate specificities of α-2,3, α-2,6, and α-2,8 linked sialic acids, respectively (Li and McClane, 2018). Neu5Ac2en has been used as the template for designing inhibitors against *C. perfringens* and *V. cholera*. This included an azido group modification at the C9 or C5 position to increase selectivity for bacterial over human SAs (10) (Khedri et al., 2012). These compounds displayed weak inhibition, but were improved in more recent work combining the aforementioned C2, C3-difluoro additions to produce a covalent inhibitor (11) (Li et al., 2019). Conjugation of a small peptide (1-4 residues) to the C9 position of Neu5Ac2en *via* a triazole linkage has been attempted and showed high selectivity for *V. cholerae* SA, with low micromolar IC_50_ values (12) (Slack et al., 2018).

*S. pneumoniae* expresses two SAs, NanA, and NanB (again family GH33), both of which have been implicated in a synergistic relationship during coinfection with influenza, potentiating, and exacerbating the effects of severe respiratory illness (McCullers and Rehg, 2002; Walther et al., 2016). This motivated investigation into the susceptibility of the bacterial SAs to the drugs oseltamivir and zanamivir, as well as two non-carbohydrate scaffold inhibitors, artocarpin, and katsumadain A (Walther et al., 2016). Only the non-carbohydrate inhibitors had the ability to inhibit both influenza and *S. pneumoniae* SAs, enabling disruption of this synergism. Other non-carbohydrate scaffolds including a Curcumin derivative, Diplacone (a naturally occurring flavanoid), and the commonly used buffering agent 2-CHES, have also been explored as compounds with potential inhibitory activity toward bacterial SAs (Lee et al., 2014; Lee et al., 2017; Kim and Park, 2018). The aforementioned α-sulphonate modifications to the anomeric carbon of Neu5Ac have also been shown to be effective against both *S. pneumoniae* and *C. perfringens* SAs (13) (Vavricka et al., 2017).

A handful of pathogenic bacteria express STs as virulence factors to produce molecular mimics of mammalian glycans, thus evading the host immune system by adding terminal sialic acid residues to their lipooligosccharides. For example, two pathogens of the genus *Neisseria* (*N. meningitidis* and *N. gonorrhoeae*) encode crucial poly-STs (family GT38) that adds α-2,8 and α-2,9 linked sialic acid chains to the extracellular capsule of these Gram-negative bacteria, mimicking mammalian poly sialic acid glycans (Bhattacharjee et al., 1975). *N. meningitidis* synthesizes Neu5Ac *de novo*, whereas *N. gonorrhoeae* scavenges the mammalian nucleotide sugar donor (Smith et al., 1995; Vogel et al., 1996). *Haemophilus influenzae* uses an α-2,3 ST which is essential for virulence in otitis media infections (Bouchet et al., 2003). Several groups have published structures of *Campylobacter jejuni* and *Pasturella multocida* STs, among other prokaryotes, however limited examples exist of inhibitor design against bacterial STs (Figure 2B) (Chiu et al., 2007; Ni et al., 2007). The general purpose ST inhibitor CMP-3F_ax_-Neu5Ac (CMP-Neu5Ac with an axial fluorine at C3 of sialic acid) (14) displays moderate inhibitory strength toward bacterial STs, with a *K*_i_ of ∼25 µM (Ni et al., 2007). Kumar *et al.* attempted to discard the scissile phosphate in favour of a selection of uncharged amide-, sulphonamide- and triazole-linked compounds, incorporating Neu5Ac, Neu5Ac2en and substituted benzene moieties. Unfortunately, these compounds all displayed relatively low inhibition. Swapping Neu5Ac for a benzene group has also been integrated in the design of fluorescent probes targeting STs, with several of these probes achieving nanomolar inhibition against *P. multocida* ST, including the non-fluorescent precursor scaffold (15) (Preidl et al., 2014).

### Trypanosome Trans-Sialidase Inhibitors

Parasitic protozoans of the *Trypanosoma* genus are responsible for extremely debilitating and life-threatening illnesses—African sleeping sickness and Chagas disease—for millions of people in less economically developed countries (World Health Organisation, 2020a; World Health Organisation, 2020b). These single-celled organisms spread through the bite of an insect vector and result in circulation of the parasite in the host’s blood (Radwanska et al., 2018). Ordinarily, such foreign material would be suppressed by the immune system, however, trypanosomes encode a crucial virulence factor (a trans-SA, belonging to family GH33) which is partly responsible for evasion from this system (Nardy et al., 2016). This enzyme is a glycoside hydrolase; however, the enzyme promotes nucleophilic attack by a glycan rather than a water molecule, and thus acts as a synthetic enzyme. The trans-SA cleaves sialic acids from host cells and covalently attaches them to terminal β-galactose acceptor molecules on the surface of the parasite, effectively masking itself in pseudo-host tissue. The inability to clear this parasite results in the establishment of chronic infection. The absence of a trans-SA in humans has established it as a promising drug target.

It is advantageous to target both the donor and acceptor sites of the trans-SA to ensure inhibition (Buschiazzo et al., 2002). To this end, a group of unusual triazole linked sialic acid and galactose disaccharides were devised in one study, resulting in a lead compound with µM potency against *T. cruzi* trans-SA (16) (Campo et al., 2012). More recently, multivalent sugar syntheses were attempted to find new trypanosome trans-SA inhibitors, with the most successful compound (17, a tetra-sialic acid conjugate) exhibiting 99% inhibition at 1 mM and an IC_50_ of 450 µM (Figueredo et al., 2019). Unfortunately, the inclusion of multiple sugars is likely to give poor absorbance and low bioavailability, highlighting the need for further research.

A wealth of other non-Neu5Ac-derived inhibitors have been designed and discovered recently which target the trans-SA. This included a search of 3180 FDA approved drugs with hopes for repurposing, benzyl β-lactoside analogues and a fragment-based screening approach based on aryl propionic acid (Lara-Ramirez et al., 2017; Kashif et al., 2018; Morrone-Pozzuto et al., 2019). One particular notable compound is a pthaloyl derivative with a predicted *K*_d_ of 7.2 nM (Neu5Ac2en has a predicted *K*_d_ of 1.9 µM) and close to 90% trypanocidal activity, which is comparable with current drugs on the market. This compound is predicted by virtual screening to use the naphthalene and indoline rings to make extensive contacts with surrounding hydrophobic residues in the active site, although this needs to be validated experimentally (Kashif et al., 2018).

### Human Sialidase and Sialyltransferase Inhibitors

In the body, STs and SAs are responsible for the addition and removal of sialic acids, respectively, from the termini of glycans presented on the surface of cells. The equilibrium between the opposing roles of these enzymes allows remodelling of the extracellular glycans and is important in many diseases (Pearce and Heinz, 2015). Four SA isozymes are encoded in the human genome termed NEU1—4 (belonging to family GH33), which differ in cellular expression and localization (Lipničanová et al., 2020). Unfortunately, NEU2 is the only human SA with its structure solved, due to the inability to grow adequate crystals of the membrane bound NEU1, 3, and 4 (Chavas et al., 2005). The roles of these SAs have been implicated in various studies to be important in cancer, atherosclerosis, diabetes, and neurodegenerative diseases (Dridi et al., 2013; Shiga et al., 2015; Zhang et al., 2019). For example, NEU3 has been implicated in the hydrolysis of sialic acid from GM3 (a common sialic acid-containing glycosphingolipid). This desialylation allows GM3 to become a ligand for the EGFR receptor and increases ERK and AKT phosphorylation, all of which have strong links to various cancers and tumorigenesis (Bonardi et al., 2014; Mozzi et al., 2015). Excessive sialylation caused by deficient levels of NEU1 may cause abnormal accumulation of amyloid precursor protein, which is known to initiate the formation of fibrils and plaques of Amyloid *β* (A*β*) and Tau proteins in Alzheimer’s disease (Annunziata et al., 2013). Furthermore, the accumulation of A*β* and Tau fibrils has recently been linked to increased desialylation of microglial cells, leading to enhanced phagocytosis of healthy neurons and further propagates the negative effects on memory (Allendorf et al., 2020; Puigdellívol et al., 2020). Minami et al. showed that the upregulation of SA activity improves hippocampal memory processing, which may provide a potential treatment for neurodegenerative diseases (Minami et al., 2017).

Although many of the aforementioned inhibitors targeting pathogens display weak inhibition of the human SAs, the lack of potent and highly specific inhibitors to aid research has hindered further progress in understanding the roles played by SAs in diseases. The most promising recent efforts to produce a human SA inhibitor built on previous work involving modification of Neu5Ac2en at C9 (Albohy et al., 2013). The best compound included a large biaryl moiety linked by a triazole at C9 of Neu5Ac2en (18), which conferred 15-fold higher selectivity for human NEU3 than the compound with just the triazole alone, and had a high nanomolar IC_50_ value (Guo et al., 2018a). The same group has also recently developed highly selective NEU1 and NEU2 inhibitors with a range of C4, C5, and C9 additions (Guo et al., 2018b). To date, these compounds represent the best tools available for research to elucidate the roles of human SAs in disease.

Humans also express 20 different, but evolutionarily related, STs, separated into three classes based on the position of the linkage between sialic acid and the rest of the glycan (α-2,3, α-2,6, or α-2,8; all belong to family GT29) (Tsuji et al., 1996; Harduin-Lepers, 2010; Lombard et al., 2014) Altered cell sialylation and upregulation of STs have been observed in many cancers and the development of metastases, including oral (Vajaria et al., 2018), liver (Dall’Olio et al., 2004), breast (Picco et al., 2010), stomach (Cebi et al., 2016), cervical (López-Morales et al., 2009) and ovarian cancers (Wang et al., 2005), among others. Using a genome-wide association study, downregulation of a polysialyltransferase (ST8-Sia-II) was found to be a potential susceptibility gene for several mental illnesses such as bipolar disorder and schizophrenia (McAuley et al., 2012). ST6-Gal-I has been heavily studied in relation to cancer [reviewed recently by Garnham et al. (2019)], with multiple studies correlating its expression to increased aggressiveness of tumours and metastases. In one instance, the death receptor Fas, which plays a key role in apoptosis, was found to be a target of abnormal sialylation by ST6-Gal-I. This appeared to downregulate the apoptotic effects of Fas, resulting in diminished activation of caspases 3 and 8, thus contributing to the “Resisting Cell Death” hallmark of cancer (Hanahan and Weinberg, 2000; Swindall and Bellis, 2011). Furthermore, the heightened tumour cell surface sialylation is an important characteristic of resistance to certain cytotoxic anti-cancer drugs (vincristine and nilotinib), suggesting that inhibition of sialyltransferases may be an effective method of re-sensitizing cancerous cells to such drugs (Parameswaran et al., 2013).

Only a handful of human ST structures exist, due to difficulties successfully crystallizing these enzymes, as they are often membrane bound proteins. The first ST structure was reported in 2013 (Kuhn et al., 2013; Volkers et al., 2015; Harrus et al., 2020). This has no doubt hindered the structure-based design of potent ST inhibitors when compared to the viral SA inhibitors. Human ST inhibitors that mimic the natural substrate are composed of three components which may be modified: the cytosine base, phosphate and sialic acid. The general-purpose ST inhibitor CMP-3F_ax_-Neu5Ac (15) displays a *K*_i_ in the low µM range against hST6-Gal-I (the most studied and targeted human ST) (Burkart et al., 2000). Skropeta and Yu’s research group have improved this drastically, publishing several papers recently to highlight tighter binding inhibitors. Many of these included α-hydroxyphosphonates in place of the carboxyl C1 as transition state analogue mimics, with later compounds also including uncharged carbamate and triazole linkers to improve potential pharmacokinetic properties (Skropeta et al., 2004; Montgomery et al., 2016; Montgomery et al., 2017). The most recent inhibitors designed by this group incorporate a benzene scaffold in place of the sialic acid (similar to the bacterial ST inhibitors) and also exchange the cytosine for a 5′-deoxy-uridine ring (19). The current lead compound from this group is a phenoxy-substituted compound (20) with a 19 nM *K*_i_ value against hST6-Gal-I. Other groups have also developed potent inhibitors of hST6-Gal-I, with simple benzene amide linked compounds intending to mimic the double bond character of the oxocarbenium transition state (21, *K*_i_ = 16 nM), cyclopentane scaffold compounds in place of the sialic acid, similar to peramivir (22, *K*_i_ = 28 nM), and the aforementioned fluorescent probe (23, *K*_i_ = 9 nM) (Li et al., 2015b ; Preidl et al., 2014; Guo et al., 2017).

Alternative methods of inhibition have been explored such as bisubstrate inhibitors (which aim to mimic the product of the ST by incorporating two or more sugars linked by thioethers or a phosphate), swapping the cytosine for 5-fluoro uracil or adenine, or modifying the cytosine with a methyl group (Hinou et al., 2002; Nakahara et al., 2004; Izumi et al., 2005; Lee et al., 2006; Miyazaki et al., 2008). A major hurdle with many glycosyltransferase inhibitors is their poor adherence to criteria for a drug candidate, with a high molecular weight, an excessive number of hydrogen bond donors/acceptors and sometimes the presence of a negative charge (Lipinski, 2004). Metabolic inhibitors of STs offer a promising solution, hijacking the cell’s usual synthetic routes to create the desired inhibitors *in vivo*, thus bypassing the need to sacrifice potency for adequate pharmacokinetic properties. However, further research is necessary to validate this method and assess the selectivity of the inhibitors for a particular enzyme (Rillahan et al., 2012). It should also be noted that the inhibition of ST8-Sia-III in the brain of mice impairs their procedural long-term memory, so characterizing the specificities of inhibitors targeting STs should be a future area of research (Rinflerch et al., 2013).

Although beyond the scope of this review, there are natural products, or natural product-like, compounds that inhibit SAs and STs. For example, soybean derived soyasaponin has been shown to inhibit cancer metastasis *in vivo*, which inspired the synthesis of lithocholic acid analogues. One cell permeable analogue attenuated α-2,3-sialylation on cell surface glycans, and was demonstrated to suppress metastasis of lung cancer cells by inhibiting modification of integrin-β1, thus impacting the FAK/paxillin/Rho signalling pathways (Chen et al., 2011). A more extensive review of other classes of ST inhibitors can be found in an article by Wang et al. (2016).

## Concluding Remarks

This review has displayed the breadth of novel SA and ST inhibitors currently being developed, particularly highlighting the recent iterative successes of using the Neu5Ac2en and Neu5Ac frameworks for new inhibitor design and concepts. The presence of SAs and STs across the domains of life and viruses highlights the need for selective and potent compounds. Structural, mechanistic, and computational information is essential to guide this design process. Several challenges exist for ST inhibition: not only in obtaining protein for the necessary *in vitro* studies, but also if incorporation of a negative charge and several polar groups in CMP-sialic acid mimics will be conducive to cell uptake and oral bioavailability. The critical roles that STs play in the progression of some human cancers, however, means research in this area is of great importance. Whilst there have been drugs in the clinic targeting viral SAs for over 2 decades, the need for new inhibitors is critical due to the ever-looming threats of pathogenic resistance and pandemics. The improvement of existing compounds presented here, and success in the design of novel compounds, will ensure preparedness in the face of these future challenges.
